# Efficacy and safety of the third-generation chloroethylnitrosourea fotemustine for the treatment of chemorefractory T-cell lymphomas

**DOI:** 10.1111/j.1600-0609.2011.01683.x

**Published:** 2011-12

**Authors:** Gaetano Corazzelli, Ferdinando Frigeri, Manuela Arcamone, Luigi Aloj, Gaetana Capobianco, Cristina Becchimanzi, Emanuela Morelli, Francesco Volzone, Gianpaolo Marcacci, Filippo Russo, Rosaria De Filippi, Secondo Lastoria, Antonio Pinto

**Affiliations:** 1 Hematology-Oncology and Stem Cell Transplantation Unit, Istituto Nazionale Tumori, Fondazione ‘G.Pascale’IRCCS, Naples; 2 Nuclear Medicine Unit, Istituto Nazionale Tumori, Fondazione ‘G.Pascale’IRCCS, Naples; 3 Department of Cellular and Molecular Biology and Pathology, Faculty of Biotechnological Sciences, Federico II UniversityNaples, Italy

**Keywords:** T-cell lymphomas, fotemustine, nitrosourea, salvage therapy, Sezary syndrome

## Abstract

Patients with recurring T-cell non-Hodgkin lymphoma (T-NHL) are incurable and candidate for investigational agents. Here, we report on five patients with T-NHL refractory to multiple chemotherapy lines, including in all cases alkylators and gemcitabine, who received the third-generation chloroethylnitrosourea fotemustine at a dose of 120 mg/m^2^ every 21 d, up to eight courses. Median actual dose intensity was 79%; toxicity was manageable and mainly hematological. One complete remission, one partial remission, two protracted disease stabilization, and one transient, minor response were achieved. Time to progression ranged from 48 to 240+ d. This is the first evidence ever reporting the activity of fotemustine in end-stage T-NHL. Formal studies with this agent are warranted in T-cell malignancies.

Non-Hodgkin lymphomas arising from mature post-thymic T cells (T-NHL) encompass biologically heterogeneous malignancies with a dismal prognosis (1, 2). Patients with histotypes such as peripheral T-cell lymphoma not otherwise specified (PTCL-NOS), kinase-negative anaplastic large cell lymphoma (ALK-negative ALCL), angioimmunoblastic T-cell lymphoma, and Sezary syndrome (SS) experience early disease recurrence with 5-yr overall survival rates rarely exceeding 40%, also because of the lack of effective salvage strategies (1–3). While the biologic basis of T-NHL aggressiveness is under extensive research, investigation into novel active agents remains a critical need, especially in the setting of stem cell transplantation ineligibility/failure and end-stage disease (2, 4).

Fotemustine is a third-generation chloroethylnitrosourea displaying a significant clinical efficacy in highly chemoresistant cancers such as disseminated melanoma and primary or metastatic brain tumors (5–9). While second-generation nitrosoureas have been incorporated into standard and high-dose salvage regimens for NHL, including T-cell subtypes (10–13), the activity of fotemustine in lymphoma has been poorly explored, despite its lower hepatic and renal toxicity, as compared to congener agents, and several more favorable pharmacodynamic properties (14, 15). First, the presence of a phosphoalanine carrier group renders fotemustine more lipophilic because its octanol/water partition coefficient results within the optimal lipophilicity interval, differently from both carmustine and lomustine (16, 17). Second, preclinical studies on rat and human tissues have shown that intracellular penetration of fotemustine is superior to that of carmustine (18, 19). Third, studies have documented that fotemustine is cleared 2–5 times more slowly than carmustine from tumor tissues (17–19). The higher lipophilic properties, the more efficient tissue distribution, and slower elimination of fotemustine from tumor tissues, as compared to other nitrosoureas, might provide a preclinical rationale for testing this drug in chemorefractory lymphomas.

Because normal and malignant T cells express a low level of *O*^6^-alkylguanine-DNA alkyltransferase (AGT) and of *O*^6^-methylguanine-DNA methyltransferase (MGMT) (20, 21), two DNA repair enzymes implicated in cellular resistance to nitrosureas, and specifically to fotemustine (22–25), we speculated that alkylator-refractory T-NHL could represent a proper setting to explore the potential clinical activity of this newer nitrosourea derivative. Here, we report the first evidence ever that fotemustine is able to induce substantial clinical responses in patients with post-thymic T-cell malignancies.

## Methods

Five consecutive patients (three men, two women; age range, 54–77 yr) with biopsy-proven recurrent T-NHL [ALK-negative ALCL (*n* = 2), PTCL-NOS (*n* = 1), and SS (*n* = 2)] were accrued into a named-patient program with single agent fotemustine. Treatment consisted of fotemustine 120 mg/m^2^, as a 1-h intravenous infusion, at day 1 every 3 wk until progression, unacceptable toxicity, or a maximum of eight administrations. The program was approved by the Internal Pharmaceutical Review Board, and after obtaining written informed consent from patients, fotemustine (Muphoran®, S10036; Servier, Neuilly-sur-Seine, France) was purchased from Italfarmaco S.p.A. (Milan, Italy). Within 2 wk prior to treatment, patients underwent a complete disease staging including bone marrow (BM) biopsy, computer-assisted tomography (CT), ^18^F-fluoro-deoxy-glucose (^18^F-FDG) positron emission tomography (PET), and echotomographic assessment of superficial disease sites. Skin involvement was assessed through the modified Severity Weighted Assessment Tool (mSWAT) (26). Levels of soluble interleukin-2 receptor (sIL-2R) were determined on cryopreserved serum samples obtained prior to fotemustine treatment. Primary prophylaxis with trimethoprim-sulfamethoxazole and valacyclovir was mandatory, while granulocyte colony-stimulating factor recommended only in case of grade 3/4 neutropenia. Response evaluation was planned every two courses, interim restaging after four courses, and final assessment at the completion of treatment, according to the International Workshop (27) and to ISCL/EORTC criteria (28).

## Case report

Baseline clinical features and treatment outcomes for all five patients are summarized in Tables 1 and 2, respectively. All were stage IV disease; most of them presented with a poor performance status and had received three prior lines of therapy [CHOP or CHOP-like chemotherapy (*n* = 3), gemcitabine-based regimens (*n* = 5), platinum and ifosfamide containing regimens (*n* = 4), newer agents including vorinostat (*n* = 1), bortezomib (*n* = 2), and alemtuzumab (*n* = 1)]. The treatment was given on an outpatient basis, and the related complications never required hospitalization. A total of 28 infusions of fotemustine were delivered. Overall, treatment duration was 8–28 wk, and the cumulative dose ranged from 280 to 880 mg/m^2^, with a median dose intensity [defined as actually delivered dose (mg/m^2^/wk) divided by the planned dose (mg/m^2^/wk)] of 79% for the first four courses. A dose reduction of fotemustine to 75% was needed, after 1-wk delay, in patients 2 (3rd course), in patient 3 (4th course), and in patient 4 (from course 3rd to 5th). All patients were evaluable for response, and none of them had to discontinue treatment owing to excessive toxicity: two had a major response, one complete (patient 1) and one partial (patient 4), and two others achieved disease stabilization (patients 3 and 5), while only one progressed following an early minor response (patient 2).

**Table 1:** Characteristics of refractory/relapsed patients with T-NHL prior to treatment with fotemustine

									Baseline					
									
*N*	Age/sex	Histology	Tumor cell phenotype^1^	Bone marrow involvement	Cutaneous involvement	mSWAT	Stage at entry	PS	WBC × 10^9^/L	Lymph × 10^9^/L	Hgb, g/L	Plt × 10^9^/L	LDH > UNL	sIL-2R IU/mL
1	54/M	Sezary syndrome	CD2+, CD3dim, CD4+, CD5+, CD7−, CD8−	Yes	Desquamating erythroderma, patch lesions, palmar fissuring	54	IVB^2^	1	6.18	4.3	12.3	215	No	722
2	78/M	Sezary syndrome	CD2+, CD3−, CD4+, CD5+, CD7−, CD8−	Yes	Multiple regressing nodules at posterior trunk, multiple plaques at left wrist, face, and scalp, erythematous patches at the left thigh	84	IVA^2^	2	5.95	1.7	10.4	243	Yes	3829
3	57/F	ALCL ALK-neg	CD3+, CD2+, CD30+, Alk−, CD20−, CD15−	Yes	No	–	IVB	3	10.8	0.3	9.5	89	Yes	928
4	74/F	ALCL ALK-neg	CD3+, CD30+, CD2−, Alk−, CD15−	No	Multiple infiltrating skin nodules at scalp and face	28	IVA	2	4.6	1.2	9.3	194	Yes	3127
5	77/M	PTCL-nos	CD2+, CD3+, CD4+, CD5+, CD7+	Yes	No	–	IVA	2	303	298	13.7	147	No	1678

M, male; F, female; ALCL, anaplastic large T-cell lymphoma; PTCL-nos, peripheral T-cell lymphoma not otherwise specified; sIL-2R, levels of soluble interleukin-2 receptor measured on prefotemustine cryopreserved serum samples with a sandwich enzyme-linked immunosorbent assay based on two monoclonal antibodies raised against two different epitopes of the p55 alpha-chain of the IL-2R complex (reference value: 180–570 IU/mL; Bender Medsystem GMBH, eBioscience, Milan, Italy); mSWAT, modified Severity Weighted Assessment Tool for skin assessment; PS, ECOG performance status.

1 As defined by immunohistochemistry and flow cytometry.

2 According to ISCL/EORTC (28).

**Table 2:** Pretreatment disease-status, response, and adverse events

*N*	Age/sex	Histology	Prior therapy *n* (type)	Response to prior therapy	Fotemustine *n* of courses	Best response	Time to best response (d)^1^	Time to progression (d)^2^	Status	CTCAE v3.0 toxicity
1	54/M	Sezary syndrome	3 (CHOP, gemcitabine, vorinostat)	Refractory	8	CR	150	240+	Alive in CR	G3 thrombocytopenia
2	78/M	Sezary syndrome	2 (GIFOX; VCG)	Refractory	3	PD	–	48	Died for PD	G4 anemia
										G4 infection
										G4 platelets
3	57/F	ALCL ALK-neg	3 (CHOP, DHAP, GIFOX)	Refractory	4	SD	42	86	Died for PD	G4 thrombocytopenia
										G3 febrile neutropenia
										G4 anemia
4	74/F	ALCL ALK-neg	3 (CHOEP, GIFOX, VCG)	Refractory	6	PR	54	165	Died for PD	G3 neutropenia
										G3 infection
5	77/M	PTCL-nos	2 (GIFOX, alemtuzumab)	Relapse	7	SD	68	144+	Alive in PD	None

M, male; F, female; ALCL, anaplastic large T-cell lymphoma; PTCL-nos, peripheral T-cell lymphoma not otherwise specified; CHOP, cyclophosphamide, adriamycin, vincristine, prednisone; CHOEP, cyclophosphamide, adriamycin, vincristine, etoposide, prednisone; GIFOX, gemcitabine, ifosfamide, oxaliplatin; VCG, bortezomib, cyclophosphamide, gemcitabine; DHAP, dexamethasone, cytarabine, cisplatin; CHOEP, cyclophosphamide, adriamycin, vincristine, etoposide, prednisone; CR, complete response; PR, partial remission; SD, stable disease; PD, progression of disease; CTCAE v3.0, Common Terminology Criteria for Adverse Events version 3.0.

1 From day of the 1st dose of treatment to documentation of best response.

2 From day of the 1st dose of treatment to documentation of progression.

**Figure 1:**
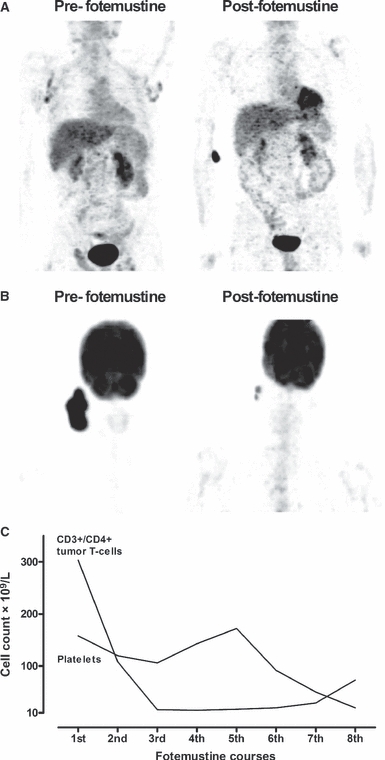
(A) Pretreatment nodal ^18^F-FDG uptakes (bilateral axillary and inguinal, intercavoaortic, lomboaortic) completely regressed after completion of fotemustine treatment in patient 1. (B) Changes ^18^F-FDG uptake of a cervical-parotid gross tumor lesion after three courses of fotemustine in patient 4. (C) The control of the leukemic (CD3+/CD4+) hypercytosis exerted by fotemustine in patient 5.

A complete response (CR) was achieved in patient 1. He had a stage IVB chemorefractory end-stage SS with high blood burden of CD4+/CD7 tumor cells, BM involvement, multicentric nodal disease (bilateral axillary and inguinal, intercavoaortic, lomboaortic) (Fig. 1A) and highly symptomatic disseminated desquamating erythroderma with patch-like skin lesions, palmar fissuring and intense, disabling itching. Skin disease completely reverted after the first two doses of fotemustine, while a residual PET-positive left inguinal adenopathy was still documented at interim restaging. A total regression of all ^18^F-FDG uptakes and a complete clearance of tumor T cells from blood and BM were documented by the end of treatment (eight courses) leading to a CR (Fig. 1A), which was maintained up to +240 d.

A partial response was achieved in patient 4. She suffered from a refractory ALK-negative ALCL with multiple skin-infiltrating nodules at the scalp and the right frontal-zygomatic area, together with a painful bulky swelling at right neck infiltrating cervical lymph nodes, parotid, and soft tissue planes. A complete regression of cutaneous involvement was achieved within 1 month from starting treatment; a maximum of 65% sharp reduction occurred, after three courses, in the sum of the products of the greatest diameters in the cervical/parotid gross lesion with an impressive decrease in the ^18^FDG uptake intensity and extension at PET scanning (Fig. 1B), as compared with baseline. Response persisted for 4 months, and thereafter, a progression was documented at day 165.

Disease stabilizations were observed in patients 3 and 5. Patient 3 had a chemorefractory ALK-negative ALCL with BM involvement and widespread nodal disease (laterocervical, retroclavear, subclavear, axillary, mediastinal, intercavoaortic, lomboaortic, splenic hilum, iliac, obturator, and inguinal) complicated by massive ascites. Treatment with fotemustine reduced nodal bulks and abrogated the need for paracentesis for about 3 months. Patient 5 had a multicentric nodal relapse of a PTCL-NOS together with a quickly onset leukemic (CD2+/CD3+/CD4+/CD5+/CD7+) hypercytosis (303 × 10^9^/L). He achieved a long-lasting control of nodal and leukemic evolution, while receiving treatment, for an overall period of 144 d, as long as T cells continued to decline in the absence of any disease-related symptoms, cytopenia, and toxicity (Fig. 1C). Subsequently, a further rise in lymphoid neoplastic cells up to 73 × 10^9^/L, paralleled by a drop in platelets count, down to below 20 × 10^9^/L, impelled for a therapeutic diversion.

Only a minor response occurred in patient 2; he had a SS with large fungating skin lesions at posterior trunk, multiple plaques at left wrist, face, and scalp, and erythematous patches at the left thigh together with nodal and leukemic (CD2+/CD3−/CD4+, CD5+, CD7−, CD8−) disease. Three fotemustine courses were delivered to this patient. Following the early rapid fainting of skin lesions, accompanied by an impressive resolution of cutaneous symptoms, he developed a full-blown disease progression shortly afterward the third course. Differently from patient 1, also affected by SS, who achieved a CR, this patient had high-burden tumoral lesions, a higher mSWAT score, and a more aggressive disease behavior as also indicated by elevated LDH values and a high serum level of sIL-2R (29, 30).

Toxicity of treatment, as assessed according to the NCI Common Terminology Criteria for Adverse Events (version 3.0), was mainly hematological. Grades 3 and 4 thrombocytopenia occurred in three patients with a total of five courses requiring transfusion support. Grade 4 anemia required transfusions in two patients for overall four courses. Febrile neutropenia requiring intravenous antimicrobial therapy complicated three courses. A destructive infection of the deep skin and tissues at back chest wall, sustained by Gram-positive cocci and anaerobic clostridia, occurred in patient 2.

## Discussion

Case reports presented herein provide the first evidence that fotemustine can achieve clinical responses in chemorefractory T-NHL. Two major responses (one complete) and two protracted disease stabilizations were recorded in four of five treated patients, all with highly unfavorable disease, recurring after alkylators and gemcitabine (i.e., two hallmarks of T-NHL therapy) and, in most cases, CHOP therapy or new agents such as bortezomib, vorinostat, and alemtuzumab. Several new agents are being actively explored in advanced T-NHL including, among others, folate antagonists such as pralatrexate, monoclonal antibodies, signaling inhibitors, and immunomodulatory agents (31). In the absence of a recognized gold standard for salvage and even upfront treatment for T-NHL (4, 31), we identified fotemustine, as a possible therapeutic option for our patients, owing to its pharmacodynamic advantages over other nitrosoureas (16–18), the low expression of AGT and MGMT in T-cell tumors (20, 21), and its favorable toxicity profile in pretreated patients (32).

Thus far, very few studies have addressed the role of fotemustine in hematological malignancies, with data published only for recurrent multiple myeloma (33, 34). More recently, we demonstrated that high doses of fotemustine (300 mg/m^2^) can be safely incorporated into an active new conditioning regimen (fotemustine, etoposide, cytarabine, and melphalan; FEAM), for autologous stem cell transplant (35). The FEAM regimen, as applied to 84 patients with lymphoma, favorably compared with the classical BEAM (carmustine, etoposide, cytarabine, and melphalan) conditioning in terms of activity and toxicity and was able to convert pretransplant PR into CR in 25 of 32 patients (78%) (35), including four of seven PTCL cases (Pinto A, unpublished observations). Interestingly, the FEAM regimen was devoid of pulmonary toxicity, as compared to the carmustine-containing BEAM, and displayed a lower rate of renal and hepatic toxicities (14, 35).

Noteworthy, fotemustine induced a fast and impressive activity on cutaneous lesions in our patients along with a rapid resolution of erythroderma and severe itching. In this respect, intravenous fotemustine may be of value in the palliative treatment for lymphoma-related refractory cutaneous symptoms. Whether this effect might be ascribed to a preferential accumulation/retention of the agent in the skin, as suggested by the highly lipophilic features (16) and significant activity of fotemustine in cutaneous melanoma (6), remains to be established.

Our patients with T-NHL were treated with a schedule differing from the treatment usually adopted for melanoma and brain tumors, i.e., 300 mg/m^2^ over an 8-wk period (100 mg/m^2^ on days 1, 8, 15 to be followed, after a 5-wk rest, by 100 mg/m^2^ at a 3-weekly interval) (6, 8). Owing to the rapid regrowth rate of tumors in our patients and the significant amount of previous myelotoxic therapy, we reasoned that fixing fotemustine dose to 120 mg/m^2^ at a 3-weekly interval could have realized a dose intensity able to control disease burden and kinetics, without a heavy load of early myelotoxicity. As a matter of fact, this allowed delivery of a sufficient number of courses at a median dose intensity approaching 80% with an acceptable hematological toxicity and without drug-related serious adverse events.

Overall, our report indicates that fotemustine is active in end-stage T-NHL and represents an agent worth of being further investigated in such disease setting. Beyond confirming our preliminary evidences, it would be of interest to study the impact of AGT expression and MGMT promoter methylation status on fotemustine activity in T-cell lymphomas. Expression of both these DNA repair enzymes was shown to predict sensitivity to fotemustine in glioma and melanoma (20–24). These studies should also be aimed at defining the optimal schedule of fotemustine for T-cell malignancies, which may differ from that used for melanoma and brain tumors.

## References

[1] Swerdlow S, Campo E, Harris N, Jaffe E, Pileri S, Stein H, Thiele J, CVardiman J (2008). WHO Classification of Tumours of Haematopoietic and Lymphoid Tissues.

[2] Dunleavy K, Piekarz RL, Zain J, Janik JE, Wilson WH, O'Connor OA, Bates SE (2010). New strategies in peripheral T-cell lymphoma: understanding tumor biology and developing novel therapies. Clin Cancer Res.

[3] Arulogun SO, Prince HM, Ng J, Lade S, Ryan GF, Blewitt O, McCormack C (2008). Long-term outcomes of patients with advanced-stage cutaneous T-cell lymphoma and large cell transformation. Blood.

[4] Foss FM (2010). Enhancing existing approaches to peripheral T-cell lymphoma. Semin Hematol.

[5] Hayes MT, Bartley J, Parsons PG, Eaglesham GK, Prakash AS (1997). Mechanism of action of fotemustine, a new chloroethylnitrosourea anticancer agent: evidence for the formation of two DNA-reactive intermediates contributing to cytotoxicity. Biochemistry.

[6] Avril MF, Aamdal S, Grob JJ (2004). Fotemustine compared with dacarbazine in patients with disseminated malignant melanoma: a phase III study. J Clin Oncol.

[7] Olson JJ, Paleologos NA, Gaspar LE (2010). The role of emerging and investigational therapies for metastatic brain tumors: a systematic review and evidence-based clinical practice guideline of selected topics. J Neurooncol.

[8] Addeo R, De Santi MS, Del Prete S, Caraglia M (2009). Fotemustine and recurrent glioblastoma: possible new opportunities for an old drug. Cancer Chemother Pharmacol.

[9] Fischel JL, Formento P, Etienne MC, Gioanni J, Frenay M, Deloffre P, Bizzari JP, Milano G (1990). In vitro chemosensitivity testing of Fotemustine (S 10036), a new antitumor nitrosourea. Cancer Chemother Pharmacol.

[10] Maurice P, Glidewell O, Jacquillat C, Silver RT, Carey R, Pas AT, Cornell CJ, Burningham RA, Nissen NI, Holland JF (1978). Comparison of methyl-CCNU and CCNU in patients with advanced forms of Hodgkin's disease, lymphosarcoma and reticulum cell sarcoma. Cancer.

[11] Palmieri G, Lauria R, Caponigro F, Pagliarulo C, Montesarchio V, Nuzzo F, Gridelli C, Bianco AR (1990). Salvage chemotherapy for non Hodgkin's lymphoma of unfavourable histology with a combination of CCNU and vinblastine. Hematol Oncol.

[12] Reimer P (2010). Impact of autologous and allogeneic stem cell transplantation in peripheral T-cell lymphomas. Adv Hematol.

[13] Dorigo A, Mansberg R, Kwan YL (1993). Lomustine, etoposide, methotrexate and prednisone (LEMP) therapy for relapsed and refractory non-Hodgkin's lymphoma. Eur J Haematol.

[14] Laquerriere A, Raguenez-Viotte G, Paraire M, Bizzari JP, Paresy M, Fillastre JP, Hemet J (1991). Nitrosoureas lomustine, carmustine and fotemustine induced hepatotoxic perturbations in rats: biochemical, morphological and flow cytometry studies. Eur J Cancer.

[15] Iliadis A, Launay-Iliadis MC, Lucas C, Fety R, Lokiec F, Tranchand B, Milano G (1996). Pharmacokinetics and pharmacodynamics of nitrosourea fotemustine: a French cancer centre multicentric study. Eur J Cancer.

[16] Levin VA (1980). Relationship of octanol/water partition coefficient and molecular weight to rat brain capillary permeability. J Med Chem.

[17] Meulemans A, Giroux B, Hannoun P, Henzel D, Bizzari JP, Mohler J (1989). Permeability of two nitrosoureas, carmustine and fotemustine in rat cortex. Chemotherapy.

[18] Meulemans A, Giroux B, Hannoun P, Robine D, Henzel D (1991). Comparative diffusion study of two nitrosoureas: carmustine and fotemustine in normal rat brain, human and rat brain biopsies. Chemotherapy.

[19] Guaitani A, Corada M, Lucas C, Lemoine A, Garattini S, Bartosek I (1991). Pharmacokinetics of fotemustine and BCNU in plasma, liver and tumor tissue of rats bearing two lines of Walker 256 carcinoma. Cancer Chemother Pharmacol.

[20] Dolan ME, McRae BL, Ferries-Rowe E, Belanich M, van Seventer GA, Guitart J, Pezen D, Kuzel TM, Yarosh DB (1999). O6-alkylguanine-DNA alkyltransferase in cutaneous T-cell lymphoma: implications for treatment with alkylating agents. Clin Cancer Res.

[21] Gallardo F, Esteller M, Pujol RM, Costa C, Estrach T, Servitje O (2004). Methylation status of the p15, p16 and MGMT promoter genes in primary cutaneous T-cell lymphomas. Haematologica.

[22] Addeo R, Caraglia M, De Santi MS (2011). A new schedule of fotemustine in temozolomide-pretreated patients with relapsing glioblastoma. J Neurooncol.

[23] Fabi A, Metro G, Russillo M (2009). Treatment of recurrent malignant gliomas with fotemustine monotherapy: impact of dose and correlation with MGMT promoter methylation. BMC Cancer.

[24] Passagne I, Evrard A, Winum JY, Depeille P, Cuq P, Montero JL, Cupissol D, Vian L (2003). Cytotoxicity, DNA damage, and apoptosis induced by new fotemustine analogs on human melanoma cells in relation to O6-methylguanine DNA-methyltransferase expression. J Pharmacol Exp Ther.

[25] Lee SM, Thatcher N, Margison GP (1991). O6-alkylguanine-DNA alkyltransferase depletion and regeneration in human peripheral lymphocytes following dacarbazine and fotemustine. Cancer Res.

[26] Olsen EA, Kim YH, Kuzel TM (2007). Phase IIb multicenter trial of vorinostat in patients with persistent, progressive, or treatment refractory cutaneous T-cell lymphoma. J Clin Oncol.

[27] Cheson BD, Pfistner B, Juweid ME (2007). Revised response criteria for malignant lymphoma. J Clin Oncol.

[28] Kim YH, Willemze R, Pimpinelli N, Whittaker S, Olsen EA, Ranki A, Dummer R, Hoppe RT (2007). TNM classification system for primary cutaneous lymphomas other than mycosis fungoides and Sezary syndrome: a proposal of the International Society for Cutaneous Lymphomas (ISCL) and the Cutaneous Lymphoma Task Force of the European Organization of Research and Treatment of Cancer (EORTC). Blood.

[29] Kitagawa J, Hara T, Tsurumi H (2009). Serum-soluble interleukin-2 receptor (sIL-2R) is an extremely strong prognostic factor for patients with peripheral T-cell lymphoma, unspecified (PTCL-U). J Cancer Res Clin Oncol.

[30] Hassel JC, Meier R, Joller-Jemelka H, Burg G, Dummer R (2004). Serological immunomarkers in cutaneous T cell lymphoma. Dermatology.

[31] Foss FM, Zinzani PL, Vose JM, Gascoyne RD, Rosen ST, Tobinai K (2011). Peripheral T-cell lymphoma. Blood.

[32] Raymond E, Haon C, Boaziz C, Coste M (1996). Logistic regression model of fotemustine toxicity combining independent phase II studies. Cancer.

[33] Dumontet C, Jaubert J, Sebban C, Bouafia F, Ardiet C, Tranchand B, Berger E, Lucas C, Guyotat D, Coiffier B (2003). Clinical and pharmacokinetic phase II study of fotemustine in refractory and relapsing multiple myeloma patients. Ann Oncol.

[34] Mangiacavalli S, Pica G, Varettoni M, Lazzarino M, Corso A (2009). Efficacy and safety of fotemustine for the treatment of relapsed and refractory multiple myeloma patients. Eur J Haematol.

[35] Musso M, Scalone R, Marcacci G, Lanza F, Di Renzo N, Cascavilla N, Di Bartolomeo P, Crescimanno A, Perrone T, Pinto A (2010). Fotemustine plus etoposide, cytarabine and melphalan (FEAM) as a new conditioning regimen for lymphoma patients undergoing auto-SCT: a multicenter feasibility study. Bone Marrow Transplant.

